# Grain boundary functions as a spin valve

**DOI:** 10.1093/nsr/nwaa074

**Published:** 2020-04-24

**Authors:** Yuichi Ikuhara

**Affiliations:** Institute of Engineering Innovation, The University of Tokyo, Japan; Nanostructure Research Laboratory, Japan Fine Ceramics Center, Japan

In practical applications, most metals and ceramic materials are used in polycrystalline form, composed of numbers of tiny single crystals called grains. The atomic arrangements at the interfaces between these grains, namely the grain boundaries (GBs), are very different from those inside the bulk part. The GB atomic structures are rather complicated and disordered, dependent on the orientations between two adjacent grains. These GBs are generally thought to be detrimental to material properties; however, recent studies have shown that they can be beneficial, and sometimes even exhibit completely different mechanical [1], electrical [2,3] and chemical properties [4,5]. In a research article published recently in NSR, Li and coauthors presented a new story of GB functionality, with a report of spin-valve magnetoresistance at a SrRuO_3_ (SRO) GB [6].

**Figure 1.**
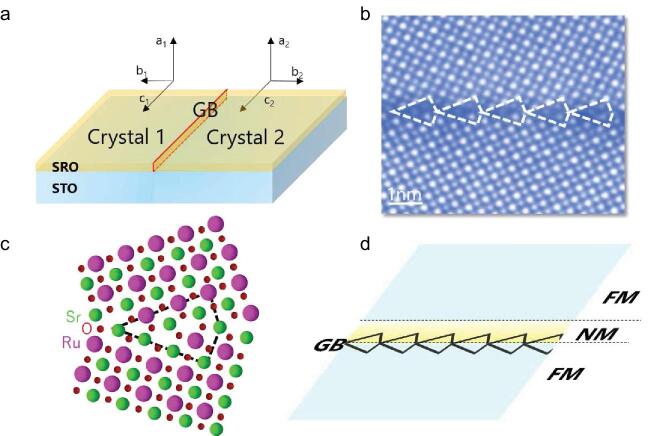
(a) Schematic image of bicrystal method used in this study. The GB structure can be controlled by controlling the crystal orientations. (b) Atomic structure of SRO GB obtained from HAADF-STEM observation (adapted from [6]) and (c) DFT calculations (courtesy of P. Gao). (d) Schematic of spin-valve structure formed near SRO GB.

An SRO model GB was designed using a bicrystal method, in which two single crystals were bonded to form a single GB (see Fig. 1a). In such cases, the GB structure can be controlled by controlling the orientations of the two single crystals. This method has been widely used for GB analysis [1–4], as the GB obtained is more suitable for electron microscopy observations and theoretical calculations than the GB inside polycrystalline materials. After fabricating a SrTiO_3_ (STO) bicrystal, SRO thin film was epitaxially grown using a pulsed laser deposition method to obtain a single SRO GB. Surprisingly, the transport measurements at the GB showed a character of spin-valve-like magnetoresistance.

The authors revealed such origin by combing aberration corrected scanning transmission electron microscopy (STEM) and density functional theory (DFT) calculations (see Fig. 1b and c). They first determined the atomic structure and chemistry using high angle annular dark field (HAADF) imaging, integrated differential phase contrast imaging and energy dispersive X-ray spectroscopy. It was found that the GB has an asymmetric core structure, which was also confirmed to be energetically favorable over a symmetric core by DFT calculations. Further analysis using DFT showed that the asymmetric GB core leads to different O octahedral distortions at two sides of the GB, with different Ru *d* orbital reconstructions. Interestingly, such structural changes drastically alter the magnetic moments near the GB; while the total magnetic moments in the region above the GB core reduced compared with those in the bulk, this changed little in the region below the GB core (see Fig. 1d). As a result, a nonmagnetic (NM) layer was formed above the GB core. As bulk SRO is ferromagnetic (FM), a layered structure of FM/NM/FM spontaneously formed at the GB, which could account for the measured transport properties.

The layered structure formed at this GB is very similar to those spin valves used for magnetic recording memory devices, which are generally realized in multilayer thin-film heterostructures with a FM/NM/FM structure [7,8]. Therefore, a single defect like GB itself might even function as a ‘device’. The present study not only opens up opportunities for novel low-dimensional device designing, but also reminds us that defects buried inside polycrystalline materials could play unexpected roles in whole device performances. Furthermore, as SRO is one of the most popular epitaxial electrode materials for complex oxide thin films [9], the existence of NM GB layer inside FM SRO might potentially change the interfacial magnetoelectric coupling in those heterostructure devices.

***Conflict of interest statement.*** None declared.
